# The Effect of Electrolyte pH and Impurities on the Stability of Electrolytic Bicarbonate Conversion

**DOI:** 10.1002/cssc.202401631

**Published:** 2024-11-12

**Authors:** Iris Burgers, Jón Jónasson, Earl Goetheer, Ruud Kortlever

**Affiliations:** ^1^ Process and Energy Department Faculty of Mechanical Engineering Delft University of Technology 2628 CB Delft, Zuid-Holland The Netherlands

**Keywords:** Bicarbonate electrolysis, Integrated CO_2_capture and conversion, Stability, pH Effects, Impurities

## Abstract

Electrolytic bicarbonate conversion holds the promise to integrate carbon capture directly with electrochemical conversion. Most research has focused on improving the faradaic efficiencies of the system, however, the stability of the system has not been thoroughly addressed. Here, we find that the bulk electrolyte pH has a large effect on the selectivity, where a higher pH results in a lower selectivity. However, the bulk electrolyte pH has no effect on the stability of the system. A decrease in CO selectivity of 30 % was observed within the first three hours of operation in an optimized system with 3 M KHCO_3_ and gap between the membrane and electrode. Single‐pass electrolyte experiments at various constant pH values (8.5, 9.0, 9.5, and 10.0), show that only at a pH of 10 the CO selectivity was stable during three hours, reaching a faradaic efficiency toward CO of only 18 % as compared to an initial 55 % at pH 8.5. Trace metal impurities present in the electrolyte were found to be the cause of the decrease in stability as these deposit on the electrode surface. By complexing the trace metal ions with ethylenediaminetetraacetic acid (EDTA), the metal deposition was avoided and a stable CO selectivity was obtained.

## Graphical abstract

The performance of bicarbonate electrolysis is largely controlled by the electrolyte inlet pH, the distance between the membrane and electrode, and the impurities present in the electrolyte. By complexing trace metal ions with ethylenediaminetetraacetic acid (EDTA), stability can significantly be improved and longer term operation can be achieved.

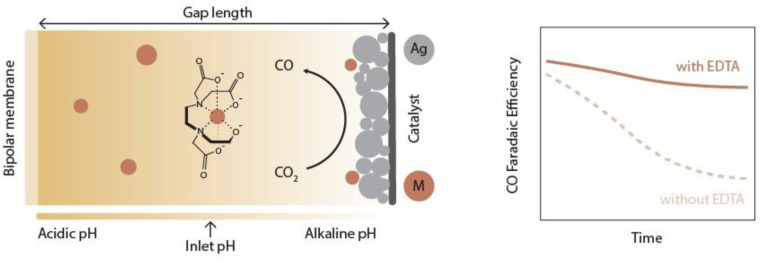

## Introduction

The electrochemical CO_2_ reduction reaction (CO_2_RR) is a promising method for using renewable electricity to convert waste CO_2_ gas into value‐added chemicals such as CO or hydrocarbons.[^1^, ^2^, ^3^] Most research focuses on gas‐fed electrolyser systems, where a pure CO_2_ gas input is required. However, these systems are still far from commercial applications, due to limitations such as low single‐pass conversion efficiency, loss of CO_2_ to carbon species, and low carbon utilization.[^4^, ^5^, ^6^, ^7^, ^8^]

To supply a pure CO_2_ gas feed to the electrolyser, a CO_2_ capture process is required to concentrate CO_2_ either from the air or from a flue gas stream. This can be achieved by a liquid absorption, such as amine based capture solvents, or solid adsorption sorbent, such as activated carbon.[^9^, ^10^, ^11^] The regeneration of the capture solvent requires an energy intensive thermal regeneration process, which reduces the economic feasibility of a CO_2_ electrolyser.[^12^, ^13^] One potential method to eliminate the energy‐intensive regeneration step is by directly using a CO_2_ rich capture solvent as input to the electrolyser. This can be achieved by using an alkaline capture solvent which forms (bi)carbonate when reacting with CO_2_. The (bi)carbonate rich solvent can be directly used in an electrolyser, where the CO_2_ is first liberated in the acidic environment near the bipolar membrane (Equations (1) and (2)) and subsequently reduced on the catalyst surface (Equation (3)).[^14^, ^15^] The main advantage of this electrolyser system is that it can provide a high concentration of CO_2_ near the catalyst surface as compared to systems using dissolved CO_2_.[^14^, ^16^] Furthermore, due to the production of OH^−^ as byproduct during CO_2_ reduction, the (bi)carbonate capture solvent is regenerated and can theoretically be recycled back to the capture column (Equation 4.
(1)

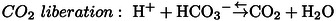



(2)

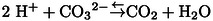



(3)

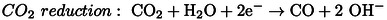



(4)

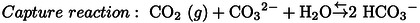




Currently, most research conducted in the field of the electrolysis of bicarbonate solutions, also referred to as bicarbonate electrolysis, has focused on improving the faradaic efficiency of the system. Berlinguette and co‐workers have published several studies on the bicarbonate electrolyser, using a Ag gas diffusion electrode (GDE) for the production of CO.[^14^, ^16^, ^17^] The electrolyser typically uses a 3 M KHCO_3_ catholyte, 1 M KOH anolyte, and a bipolar membrane to supply a constant H^+^ flux to the cathode compartment. A porous carbon support layer spray‐coated with Ag nanoparticles and Ni foam served as cathode and anode, respectively. An initial faradaic efficiency towards CO (FE_CO_) of around 40 % was reached at 100 mA/cm^2^.^[14]^

An in‐depth electrode design analysis was performed by the same group, looking into different deposition techniques such as spray‐coating, physical vapor deposition, and a combination of these two methods.^[16]^ Their optimized GDE, a combination of a 500 nm thick Ag layer, deposited using physical vapor deposition (PVD), and a spray coated Ag layer, resulted in a reported FE_CO_ of 82 % at 100 mA/cm^2^, which was confirmed by personal communication to be the initial faradaic efficiency after 5 minutes of operation.^[19]^ Furthermore, the use of a free standing porous Ag electrode was compared with a Ag GDE.^[18]^ An initial FE_CO_ of around 60 % was reached using a porous electrode at ambient conditions and 100 mA/cm^2^. Despite the decrease in performance of the porous electrode compared to the Ag GDE, a porous electrode was argued to be better than a GDE due to the higher durability and easy handling.^[18]^ Increasing the pressure up to 4 bar resulted in a significant increase in FE towards CO up to 95 % at 100 mA/cm^2^, due to the increased solubility of CO_2_.^[18]^ However, the stability of the bicarbonate electrolysis system was not discussed in detail.

Carbonate reduction, as opposed to bicarbonate reduction, using a Ag GDE was investigated by Li et al.^[15]^ The performance of a carbonate electrolyser was studied with different concentrations of K_2_CO_3_ (0.1 to 2 M) as catholyte. At a concentration of 2 M K_2_CO_3_ and current density of 100 mA/cm^2^, a FE_CO_ of approximately 30 % was obtained. Recently, Xiao et al.^[20]^ demonstrated that by physically separating the catalyst and the membrane with a thin TiO_2_ layer (25 μm) on top of the catalyst, the FE_CO_ increases from around 10 % to 46 % at 200 mA/cm^2^ for a system using a 2 M K_2_CO_3_ catholyte at a pH between 10 and 11. Similarly, a study by Lee et al.^[21]^ further investigated the effect of a spacing between the membrane and catalyst to improve the pH gradient in the system. They modelled the local pH as a function of the distance between the membrane and the electrode surface for a carbonate electrolyser. Interestingly, they found that in a zero‐gap configuration, the pH at the membrane does not reach acidic conditions. When introducing a spacing of 135 μm, the pH at the membrane decreases to around 3 at 200 mA/cm^2^, providing the right conditions for the in‐situ generation of CO_2_ inside the system.

The above mentioned results seem very promising for using a bicarbonate electrolyser in an integrated capture and conversion system. However, as mentioned before, the stability of all of these systems remains unclear and is often overlooked in the current literature. Lees et al.^[16]^ briefly discuss the decrease of the FE_CO_ over time and conduct an 8 hour experiment where the 3 M KHCO_3_ electrolyte was refreshed every three hours, showing a temporary recovery of the CO faradaic efficiency to the initial efficiency of around 40 % after refreshing the electrolyte. Furthermore, the Ag loading on the electrode before and after electrolysis measured by XRF was within 2 % difference. Additionally, they performed a control experiment in which they acidified the recirculating electrolyte by adding 4 M H_2_SO_4_ after every 2 hours of operation during an 8 hour experiment, such that the electrolyte pH remains around 8.5. In this case, the FE_CO_ does not completely recover to the initial 40 % and an overall decreasing CO selectivity is measured over time. This is explained by a depletion of the carbon present in the electrolyte. Therefore, it is suggested by Lees et al.,^[16]^ that the increase in pH and decrease in carbon concentration is the main cause for the decrease in the product selectivity over time.

This work focuses on quantifying and understanding the changes in CO selectivity in a bicarbonate electrolyser as function of time. To understand the role of the electrolyte pH on the stability of the system, several experiments with recirculated electrolytes and single‐pass electrolytes at a various bulk pH conditions were studied. The bulk pH strongly effects the overall FE_CO_, where a more alkaline electrolyte results in a lower FE_CO_. However, the bulk pH does not control the stability of the system. Instead, the deposition of trace metal ion impurities which are present in the high concentration electrolyte salt are found as the main cause for the decrease in selectivity. By complexing these trace metal ions with ethylenediaminetetraacetic acid (EDTA), the metal deposition was avoided and a stable CO selectivity was obtained.

## Results and Discussion

### Improving In‐situ CO_2_ Liberation

**Figure 1:**
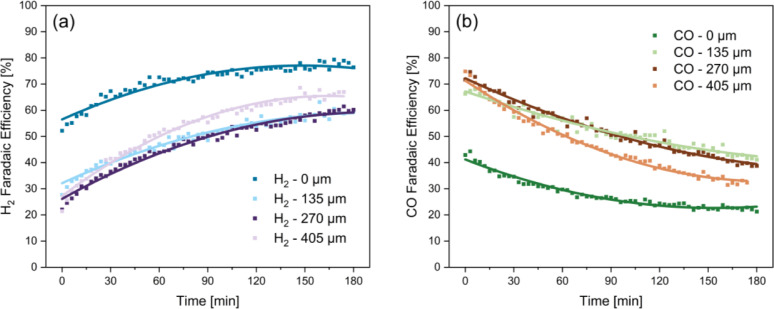
Bicarbonate electrolysis experiments at 100 mA/cm^2^ using a Ag spray‐coated cathode and recirculating 3 M KHCO_3_ catholyte (70 mL) and 1 M KOH (140 mL). Faradaic efficiencies of (a) H_2_ and (b) CO over time, for different distances between the membrane and the catalyst layer. All data points are average values of duplicate measurements with an average error of ±3.2 % and total FE of >98 %. The lines represent polynomial fitting of the data for a better representation of the stability trend over time.

Initial bicarbonate electrolysis experiments were conducted using a zero‐gap configuration and recirculating electrolytes leading to an initial FE_CO_ of around 40 % (see Figure 1b). This obtained result is similar to previously reported values.[^14^, ^15^, ^16^] To improve the in‐situ CO_2_ liberation, a spacing between the membrane and electrode was introduced, as suggested previously by Lee et al.^[21]^ for carbonate reduction, using a mixed cellulose ester (MCE) membrane. It was shown that the spacing improved the pH gradient between the membrane and the electrode, creating an acidic pH for CO_2_ liberation at the membrane, while maintaining an alkaline pH for CO_2_ reduction at the cathode. Lee et al.^[21]^ showed an optimal increase in C_2+_ selectivity when using a spacing of 135 μm in a carbonate electrolyte with a pH in the range of 10 to 11. To understand how this translates to a bicarbonate electrolysis system with a lower pH, three different gap dimensions (135, 270, and 405 μm) were tested in the bicarbonate electrolyser system (pH 8.2±2). In Figure 1 the faradaic efficiencies of H_2_ and CO for the four different conditions are presented as a function of time. The measured cell potential was 3.6±0.1 V for all experiments. We find that by introducing a spacer in between the bipolar membrane and electrode the faradaic efficiencies towards CO are significantly improved. Initially, around 70 % FE_CO_ was achieved with all three spacing dimensions, compared to only 40 % for the zero‐gap configuration. The highest reported initial FE_CO_ c is 82 %, which was achieved by using an optimized electrode consisting of a 500 nm Ag PVD layer on both sides of the electrode and a spray coated Ag layer on the side facing the membrane.^[16]^ Our results show that the use of a spacer results in a similar initial FE_CO_, while using a simple electrode preparation with significantly less Ag loading, making it more economical.

As can be seen in Figure 1, the product selectivity decreased significantly during the 3 hour experiment for all four tested spacings. For the zero‐gap configuration, a FE_CO_ of only 25 % is reached after three hours of operation. Using a spacer did not prevent the decrease in FE_CO_. The stability of the system with a gap of 135 μm and 270 μm is very comparable. When the introduced spacing is increased to 405 μm, the decrease in FE_CO_ over time is more significant as compared to all other configurations, due to the increased rate of in‐situ CO_2_ capture over the longer distance in between the membrane and electrode.[^20^, ^21^] In summary, the typically reported faradaic efficiencies in the literature are the initial values, which do not accurately represent the behavior of the system. Even though the FE_CO_ can be increased by introducing a spacer, with a spacing of 135 μm performing best, the observed significant decreases in FE_CO_ over time are undesirable for continuous operation of a bicarbonate electrolyser and should therefore be explored further.

### Longer Term Stability

**Figure 2:**
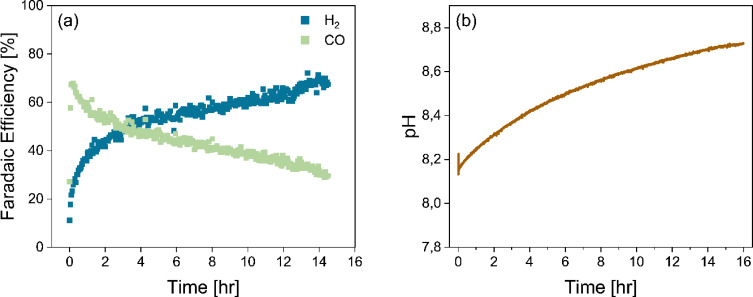
Long term stability of bicarbonate electrolyser at 100 mA/cm^2^ using a Ag spray‐coated cathode and recirculating 3 M KHCO_3_ catholyte (1 L) and 1 M KOH anolyte (0.5 L). (a) Faradaic efficiencies over time towards H_2_ and CO for a configuration with a spacing between the electrode and membrane of 135 μm. (b) The change in electrolyte bulk pH over time. All results are average values of duplicate measurements with an average error of ±3 % and total FE of >98 %.

The results obtained in the three hour bicarbonate electrolyser experiments suggest that the product distribution was reaching stability near the end of the experiments. In order to better characterize the longer‐term stability, 15 hour experiments were conducted. In Figures 2 and S3, the faradaic efficiencies of H_2_ and CO are represented as a function of time, as well as the bulk pH over time for a zero‐gap configuration and for a spacing between the electrode and membrane of 135 μm. Similar to the 3 hour experiments, recirculating electrolytes were used in the 15 hour experiments. Due to the longer duration of the experiments, the volumes of the catholyte and anolyte were increased to 1 L and 0.5 L, respectively, to ensure that the (bi)carbonate and KOH concentrations were maintained relatively constant. The pH was measured at the outlet of the electrolyser, and represents the bulk pH of the catholyte.

The results shown in Figure 2 demonstrate that the FECO does not stabilize over 15 hours of operation. The change in selectivity is larger in the beginning, and slows down near the end of the 15 hours. The pH of the 1 L catholyte increases steadily from around 8.1 to 8.7 after 15 hours of operation. The steady decrease after the first 2 hours can be related to the steady increase in pH of the bulk electrolyte. A very similar observation was made by Lees et al.,^[16]^ in which the pH increased in just 2 hours from 8.5 to 9 for a bicarbonate electrolyser using only 125 mL of catholyte and comparable electrode surface area. Similarly, at a higher pH, the FE_CO_ dropped from 40 % to 30 % faradaic efficiency. However, the larger initial change in selectivity during the first 2 hours of the experiment is not expected to be caused by the change in bulk pH, as the selectivity decrease is much faster than the measured gradual rise in pH. To confirm this hypothesis, a series of single‐pass electrolyte experiments were conducted.

### The Effect of pH on CO Selectivity

As suggested previously,^[16]^ the increase in pH and decrease in FE_CO_ suggests that the pH is affecting the selectivity of the CO_2_RR and competing hydrogen evolution reaction (HER). Therefore, a set of single‐pass catholyte experiments were conducted with a different inlet pH, while maintaining a constant 3 M K^+^ concentration. Four different (bi)carbonate electrolytes with a pH of 8.5, 9.0, 9.5, and 10.0 were evaluated in the bicarbonate electrolyser, using a gap of 135 μm at a constant current of 100 mA/cm^2^. The anolyte was recirculated, as no changes in pH were observed and this is thus not limiting the anodic reaction.

**Figure 3:**
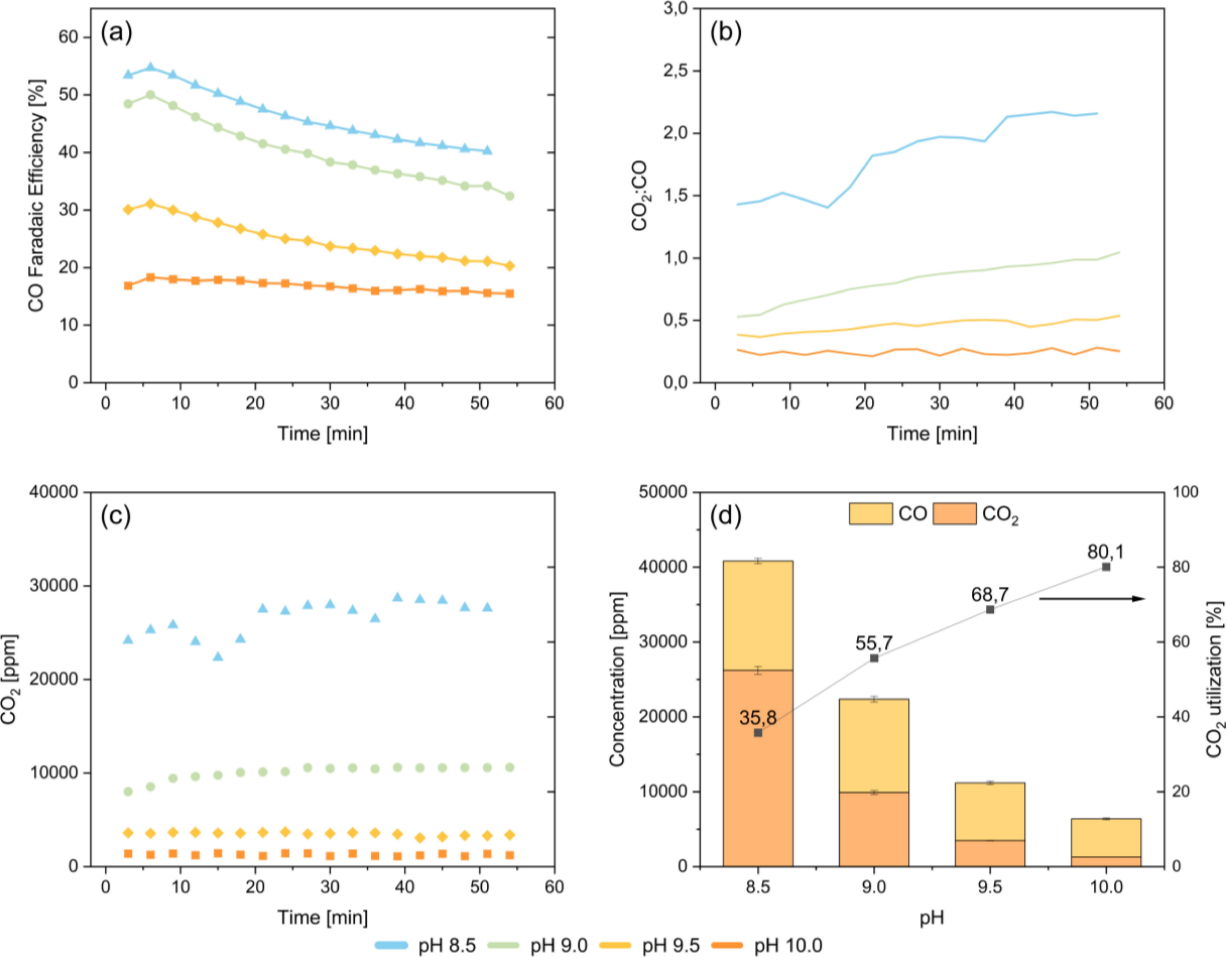
The effect of constant pH inlet conditions for a pH of 8.5, 9.0, 9.5, and 10.0 in a bicarbonate electrolyser using a Ag spray‐coated cathode at a constant applied current of 100 mA/cm^2^, and a fixed spacing of 135 μm between the membrane and the cathode. (a) Faradaic efficiencies towards CO, (b) CO_2_:CO ratio over time, (c) total CO_2_ concentration measured at outlet of the electrolyser in ppm, (d) total concentration carbon at the outlet of the electrolyser (CO products and CO_2_ unreacted) in ppm and the CO_2_ utilization ratio.

In Figure 3(a), the FE_CO_ for the four different inlet pH conditions are presented as a function of time. These results clearly demonstrate that a constant inlet pH does not result in a constant product output, indicating that there are more factors contributing to the decrease in FE_CO_ of the bicarbonate electrolyser system. It can however clearly be observed that an increase in pH decreases the selectivity towards CO production. At a pH of 8.5, the initial FE_CO_ reaches 55 %, whereas for a pH of 9.0, 9.5, and 10.0 the FE_CO_ initially reaches 50 %, 31 %, and 18 %, respectively. Interestingly, at a constant catholyte pH of 10.0, a relatively stable faradaic efficiency towards CO is observed.

At a lower pH of 8.5, the concentration of unreacted CO_2_ at the outlet is close to 30 000 ppm, as can be seen in Figure 3(c). At a pH of 9.0, the concentration significantly decreases to only 10 000 ppm. At a pH of 9.5 and 10.0, the concentration of unreacted CO_2_ in the outlet is approximately 2500 and 1300 ppm, respectively. The lower CO_2_ concentration at a higher pH is due to the higher concentration of carbonate. CO_2_ liberation from carbonate requires 2 protons instead of 1 proton required for CO_2_ liberation from bicarbonate (Equations (1) and (2)), making CO_2_ liberation from carbonate more sluggish. Furthermore, the CO_2_ reabsorption rate is higher at higher pH. These results are in line with unreacted CO_2_ concentrations reported in earlier studies[^14^, ^16^] The CO_2_:CO ratio, reported in Figure 3(b), demonstrates that unreacted CO_2_ concentration rises as CO production decreases over time. Furthermore, the cell potential was 3.6±0.1 V for all four different pH conditions and remained stable over time.

The average CO_2_ utilization ratio was calculated based on the average CO and CO_2_ concentrations measured at the outlet. The CO_2_ utilization ratio was calculated using Equation (5) and represented in Figure 3(d).
(5)

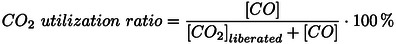




There is a very clear trend visible, showing an increased CO_2_ utilization ratio at higher electrolyte pH. A utilization of 80 % was achieved at pH 10.0, compared to only 36 % at pH 8.5. The CO_2_ utilization ratio previously reported for carbon composite electrodes using 3 M KHCO_3_ (pH~8) at 100 mA/cm^2^ is around 20 %–30 %.^[16]^ The higher CO_2_ utilization ratio stems from the increased CO_2_ reabsorption to form (bi)carbonates at higher pH concentrations, and not from the increase in CO_2_ conversion to CO.

**Figure 4:**
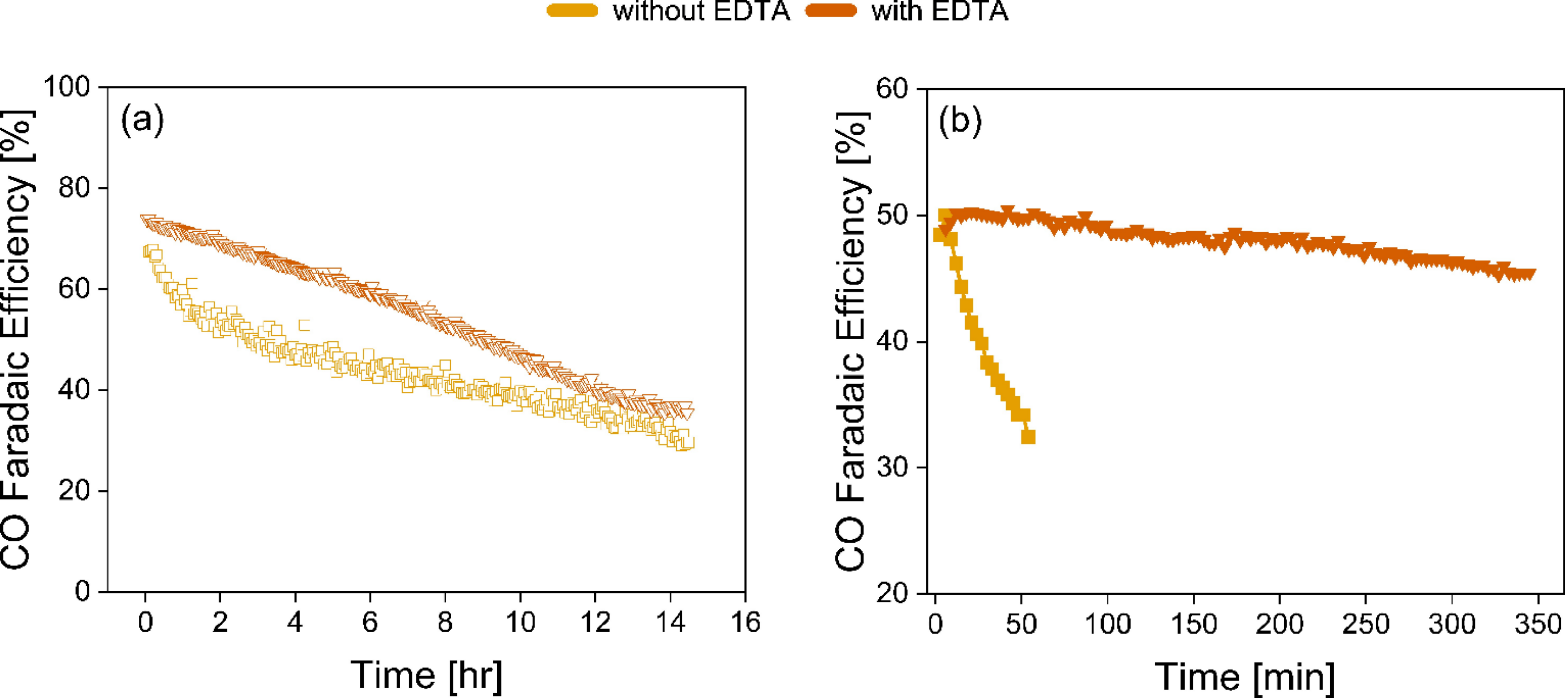
Stability experiments using 0.02 M EDTA at 100 mA/cm^2^ using a Ag spray‐coated cathode. Faradaic efficiency towards CO are shown comparing with and without the addition of EDTA for (a) longer‐term 15 hour run using recirculating electrolyte, and (b) single‐pass electrolyte at pH 9.

The results presented here contradict the hypothesis that a constant inlet pH of the catholyte will provide a constant product selectivity as suggested earlier.^[16]^ Although there is a clear dependency of the FE_CO_ on the inlet pH, with a higher pH leading to a lower FE_CO_, for all inlet pH values a similar decline in FE_CO_ over time is observed compared to the experiment with recycled electrolytes. Therefore, we hypothesize that the decline in FE_CO_ during experiments must stem from changes of the electrode. However, scanning electron microscopy (SEM) images (Figure S5) showed no visible degradation of the catalyst surface. Additionally, through inductively coupled plasma spectroscopy (ICP) analysis (Table S1), no Ag catalyst traces were found in the electrolyte post electrolysis, but very low quantities of Fe, Na and Cl were detected. Previous studies in CO_2_ electroreduction have suggested that catalyst deactivation can occur due to the deposition of trace metal ions or organic impurities in the electrolyte.[^22^, ^23^, ^24^] By complexing these trace metal ion impurities with EDTA, the metal deposition can be suppressed.^[24]^ Two control experiment using 0.02 M EDTA were performed. First, 15 hour experiments with 1 L of 3 M KHCO_3_ recirculating electrolyte, and second a single‐pass experiment using a 3 M KHCO_3_/K_2_CO_3_ catholyte at a constant inlet pH 9 for 6 hours were conducted. The results were compared to the initial measurements wherein no EDTA was used and are presented in Figure 4. For the longer term experiment a linear decrease of FE_CO_ over time is observed. The larger initial drop at the start of the 15 hour experiment in which no EDTA was added is therefore very likely related to the deposition of trace metal impurities on the surface. The total decrease of the FE_CO_ for the experiment in which EDTA was used is larger than for the experiment in which no EDTA was used. It is hypothesized that this is related to the change in pH over time, which was measured to be larger for the experiment in which EDTA was added (see Figure S6). A significant improvement in the CO selectivity over time is observed for the single‐pass electrolyte control experiment. A total decrease of 5 % FE_CO_ over 6 hours is measured. This confirms that the decline in FE_CO_ when recirculating the electrolyte is related to the pH and EDTA can significantly improve the stability of the system by removing metal trace impurities from the electrolyte and protecting the electrode active surface area. Due to the very low concentration of the impurities in the electrolyte salt (see Table S2), it is difficult to measure the impurities on the electrode surface using X‐ray photoelectron spectroscopy (XPS). Wuttig et al.^[24]^ managed to measure the impurities by using a rotating disk electrode to increase the rate of diffusion‐limited metal deposition. However, for the electrode used in this study, it is not feasible to measure the concentration of the impurities at the surface. Next to metal impurities such as Fe, other impurities such as Ca^2+^ and Mg^2+^ can also affect the stability of the electrolysis system at a larger scale. It is known from water electrolysis literature that Ca^2+^ and Mg^2+^ ions can adhere to the membrane, lowering the conductivity, or cathode surface, limiting the access to the electrode active sites.[^26^, ^27^, ^28^] A fundamental understanding of the electrode stability is highly desirable, including understanding the destabilization mechanisms for CO_2_ reduction systems, caused by electrocatalytic degradation and changes in the catalyst microenvironment evolution during long‐term operation.[^29^, ^30^, ^31^]

## Conclusions

In this study, the product selectivity of a bicarbonate electrolyser as a function of time as well as the role of the electrolyte pH has been evaluated. We observed that the product selectivity of the bicarbonate electrolyser at a fixed current density is not stable over time. Introducing a spacing between the membrane and the catalyst improves the product selectivity of the CO_2_RR towards CO, however it does not improve the stability of the system. A decrease in FE_CO_ of 30 % during the first three hours of operation is observed. Longer‐term experiments of 15 hours show a continuous decrease in the product selectivity, related to the steady increase of the recirculated electrolyte bulk pH. It is found that the bulk electrolyte pH has a large effect on the overall selectivity, where a more alkaline pH lowers the selectivity towards CO. However, the bulk electrolyte pH was found to have no effect on the stability of the system. Electrolytes of four different pH values (8.5, 9.0, 9.5, and 10.0) were tested in a single‐pass configuration. At a pH of 10.0, the product selectivity towards CO was constant over time, reaching a FE_CO_ of 18 % compared to an initial 55 % at pH 8.5. The total amount of CO_2_ liberated at higher pH conditions is significantly lower, hence less CO_2_ is available for the CO_2_RR. This does improve the CO_2_ utilization ratio of the system, which is only 36 % at a pH of 8.5 compared to 80 % at pH 10.0. Finally, a stable FE_CO_ selectivity was obtained by using EDTA to complex the trace metal ion impurities present in the electrolyte salt and prevent its deposition on the electrode surface. A significant improvement in stability was measured, with a small drop of 4 % FE_CO_ during 6 hour operation.

## Conflict of Interests

The authors declare no conflict of interest.

1

## Supporting information

As a service to our authors and readers, this journal provides supporting information supplied by the authors. Such materials are peer reviewed and may be re‐organized for online delivery, but are not copy‐edited or typeset. Technical support issues arising from supporting information (other than missing files) should be addressed to the authors.

Supporting Information

## Data Availability

The data that support the findings of this study are available from the corresponding author upon reasonable request.
