# Supplementary material for: The Effect of Electrolyte pH and Impurities on the Stability of Electrolytic Bicarbonate Conversion
Source: ChemSusChem. 2024 Nov 12;18(6):e202401631. doi: 10.1002/cssc.202401631 (PMC11912112; doi:10.1002/cssc.202401631)
Supplement: Supplementary file 1 — Supporting Information [file CSSC-18-e202401631-s001.pdf]

ChemSusChem

Supporting Information

The Effect of Electrolyte pH and Impurities on the Stability of Electrolytic Bicarbonate Conversion

Iris Burgers, Jón Jónasson, Earl Goetheer, and Ruud Kortlever*

Supporting Information to

The Effect of Electrolyte pH and Impurities on the Stability of Electrolytic Bicarbonate Conversion

Iris Burgers, Jón Jónasson, Earl Goetheer, Ruud Kortlever*

I.A.E. Burgers, J.K. Jónasson, E.L.V. Goetheer, R. Kortlever

Process and Energy Department, Faculty of Mechanical Engineering

Delft University of Technology

Delft, Zuid-Holland, 2628 CB, The Netherlands

E-mail: r.kortlever@tudelft.nl

Figure S1. Schematic of the lab-scale bicarbonate electrolyser. (a) Exploded view of flow cell including all the different components, (b) stacked electrolyser, (c) drawing of the interdigitated flow plate, all dimensions in mm. 2

Figure S2. Lab-scale bicarbonate electrolyser. (a) and (b) commercial flow cell electrolyser, (c) components used in the stack of the electrolyser: 1) End plate, 2) Gasket (1 mm thickness), 3) Ni current collector, 4) Flow pattern gasket (2x 1 mm thickness), 5) Ni foam anode (2 mm thickness) and flow distributors, 6) Bipolar membrane, 7) Gasket (200 um thickness) and Ag GDE, 8) Titanium interdigitated flow pattern and current collector, 9) Gasket (1 mm thickness), 10) End plate. 3

Figure S3. Chromatograms of (a) the first channel, measuring methane and ethylene, (b) the second channel, measuring CO_2 and (c) the third channel, measuring hydrogen and CO. 5

Figure S4. Longer term stability of zero-gap bicarbonate electrolyser at 100 mA/cm^2 using a Ag spray-coated cathode and recirculating 3 M $KHCO_3$ catholyte (1 L) and 1 M KOH anolyte (0.5 L). (a) Faradaic efficiencies over time towards H_2 and CO for a zero-gap configuration. (b) The change in electrolyte bulk pH over time. All results are average values of duplicate measurements with an average error of ± 3% and total FE of >98%. 6

Figure S5. SEM images (a) and (c) fresh electrodes, (b) and (d) post-electrolysis. Except for small cracks due to the catholyte flow through the electrode, no visual changes of the surface are visible. 6

Figure S6. The change in electrolyte bulk pH over time for the longer term stability experiment of the bicarbonate electrolyser at 100 mA/cm^2 using a Ag spray-coated cathode and recirculating 3 M $KHCO_3$ catholyte (1 L) and 1 M KOH anolyte (0.5 L). 8

Table S1. ICP results for the electrolyte of the longer-term electrolysis runs (3 hours and 15 hours), presented in ppm. 7

Table S2. List of contaminants present in the $KHCO_3$ (batch BCCL2557) and K_2CO_3 (batch BCCL0256) used.[1, 2] 7

Experimental Section

Bicarbonate Electrolysis

A commercial ElectroCell flow cell (Micro Flow cell 10 cm^2 MFC30010) was used to perform the bicarbonate electrolysis experiments. An exploded view of the cell and the different components can be seen in Figure S1. A nickel foam (2 mm thickness, ElectroCell) was used as the anode and a bipolar membrane (Fumasep FBM-PK) was used to separate the cathode and anode. The bipolar membrane (BPM) was stored in 1 M NaCl and reused multiple times, but replaced regularly. The Ni foam was stored in water and sonicated for 5 minutes prior to experiments, and replaced regularly as well. A 3 M $KHCO_3$ (99.97%, Sigma Aldrich) solution was prepared in 1 L stock solutions at a time and the pH measured. For the single-pass pH experiments, different pH buffer solutions of $KHCO_3$ and K_2CO_3 were mixed to obtain the required pH. The anolyte used was 1 M KOH (>85%, Sigma Aldrich). A mixed cellulose ester (MCE, 8 μm pore diatemeter, MF-Millipore) membrane was used as a spacer between the membrane and the cathode. The membrane was wetted with MilliQ water after which it was stored in the catholyte solution for about 5 minutes to ensure full saturation prior to assembling the electrochemical cell.

To assemble the cell, the EPDM sealing gaskets supplied by ElectroCell were used, as well as a thinner silicon gasket for the cathode. On the anode side, a Ni current collector was used whereas on the cathode side a Ti interdigitated flow plate was used as current collector. The interdigitated flow plate had 1 mm wide flow channels and bridges (see Figure S1(c)). The electrode stack was closed evenly by 6 bolds, using a torque wrench at 3 Nm. In Figure S2, all components used in the bicarbonate flow cell are depicted.

For the 3 hour experiments, a catholyte volume of 70 mL and anolyte volume of 140 mL were used. The overnight 15 hour runs used 1000 and 500 mL of catholyte and anolyte respectively. For the single-pass experiments, the anolyte volume was kept at 140 mL and the catholyte volume was increased to 5 L. An inline pH probe (InPro3250i with Transmitter M300 Water 2-channel, Mettler) was installed at the outlet of the bicarbonate electrolyser, to measure the output pH continuously during an experiment. A peristaltic pump (BT100-3J Basic Peristaltic Pump, Darwin Microfluidics) was used in combination with 2 pump heads (YZ1515X-B, Darwin Microfluidics) and silicon tubing (#25, Darwin Microfluidics). A flow rate of 50 mL/min was used for both electrolytes. A potentiostat (SP-200, Biologic) was used as the current supply, using the EC-lab software. A constant current of 500 mA was applied to the cathode, which corresponds to 100 mA/cm^2 as the active area of the cathode is 5 cm^2.

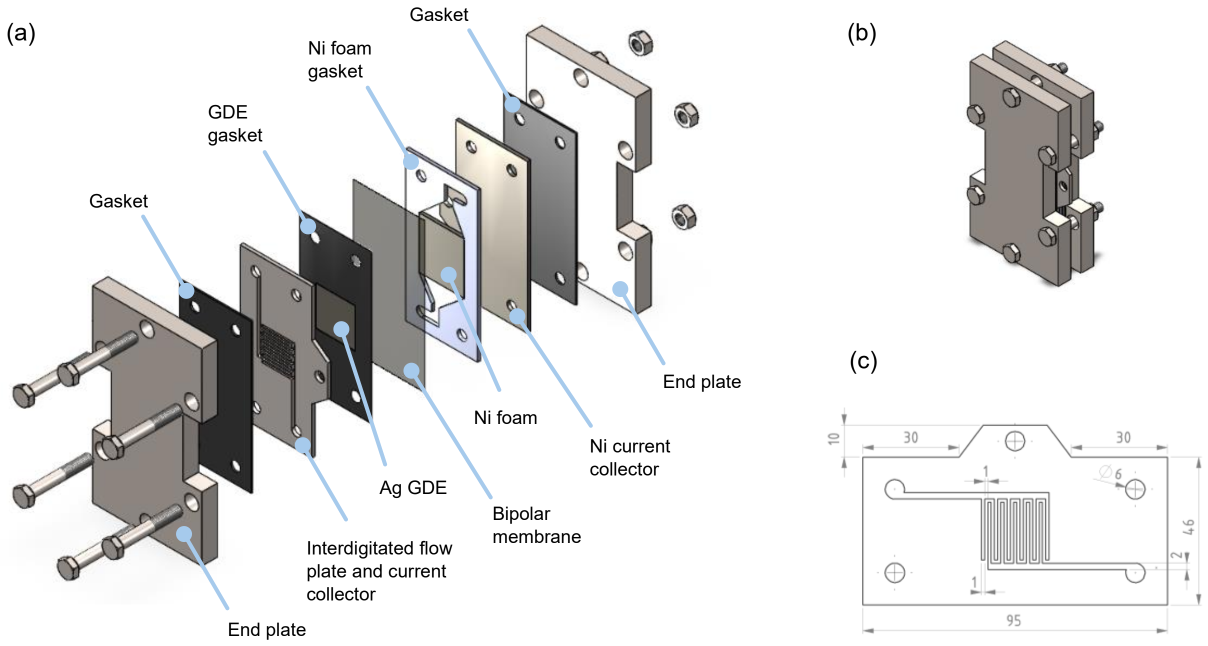

Figure S1. Schematic of the lab-scale bicarbonate electrolyser. (a) Exploded view of flow cell including all the different components, (b) stacked electrolyser, (c) drawing of the interdigitated flow plate, all dimensions in mm.

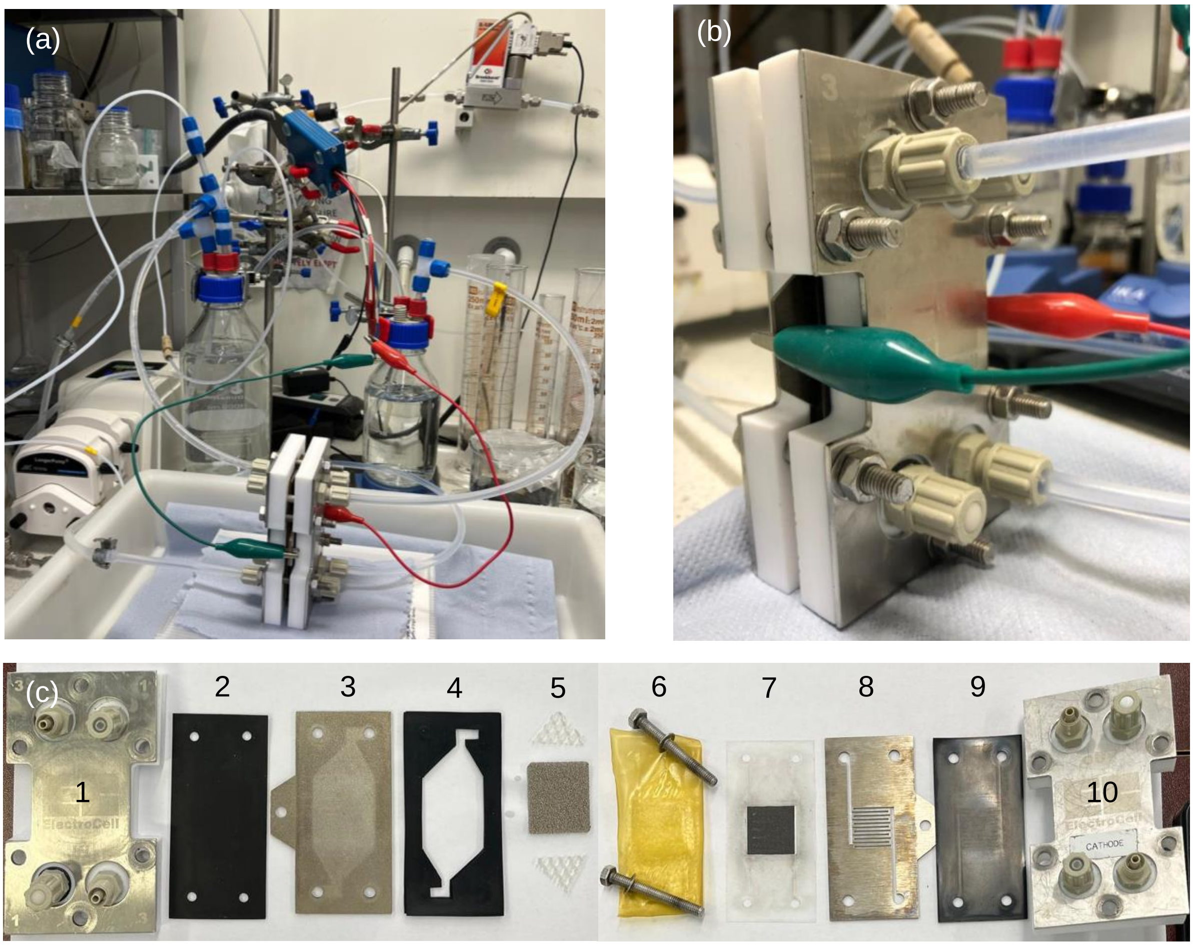

Figure S2. Lab-scale bicarbonate electrolyser. (a) and (b) commercial flow cell electrolyser, (c) components used in the stack of the electrolyser: 1) End plate, 2) Gasket (1 mm thickness), 3) Ni current collector, 4) Flow pattern gasket (2x 1 mm thickness), 5) Ni foam anode (2 mm thickness) and flow distributors, 6) Bipolar membrane, 7) Gasket (200um thickness) and Ag GDE, 8) Titanium interdigitated flow pattern and current collector, 9) Gasket (1 mm thickness), 10) End plate.

Electrode preparation

The Ag catalysts were prepared by spray-coating (Custom Micron Absolute Precision, Iwata) a Ag nanoparticle (20-40 nm, Thermo Fisher Scientific) ink onto a carbon support layer (Sigracet 39BB, Fuelcell Store). The carbon support layers were cut into pieces of 25 x 30 mm to allow extra space to hold the electrode in place during spray-coating. An area of 25 x 25 mm was spray-coated and the excess material was cut off post electrode preparation. Prior to spray-coating, the carbon material was sonicated in acetone and MilliQ water subsequently, for 10 minutes each. The electrodes were dried on a hot plate (covered with aluminium foil) at 90 °C for about 10 minutes until completely dry. The dry electrodes were weighed to determine the pre-spray-coating weight. Before spraying, the heat of the hotplate was reduced to 70 °C to ensure rapid evaporation of the isopropanol and curing of the Ag-Nafion layering.

For the ink, 84 ± 2 mg of Ag nanoparticles (20-40 nm, Thermo Fischer Scientific) were mixed with 8 mL of isopropanol (VWR Chemicals) and 80 µL of Nafion binder (Nafion 1100W, 5 wt% in water and propanol, Sigma Aldrich). The ink was sonicated for 30 minutes in an ultrasonic bath (2800 Ulstrasonic cleaner 2.8L, Branson) and used within 30 minutes of preparation. Using a spray gun (Custom Micron Absolute Precision, Iwata) connected to a N_2 gas line at a pressure of 0.6 bar, the ink was dispersed onto the carbon papers. Before using the ink, the ink was vortexed for about 10 seconds. A total of 2 mL of ink was pipetted into the cup of the spray gun, which was used to spray the first layer onto 2 electrodes. This was repeated again with 2 mL of ink, to make two electrodes with a loading of 1 ± 0.3 mg Ag/cm^2 and 4wt% Nafion. The prepared electrodes were kept on the hot plate for 10 more minutes to ensure all isopropanol had evaporated, after which they were weighed again to measure the loading.

Gas product analysis

The gas products formed were measured inline using a gas chromatograph (Compact GC 4.0, Interscience). The GC was equipped with two thermal conductivity detectors and one flame ionization detector. Samples were measured at 3 minute intervals during the experiments. Argon was used as flushing gas, which was purged in the head space of the catholyte vessel at a constant flow rate of 100 ml/min using a mass flow controller (Bronkhorst). The outlet gas stream of the catholyte vessel passed through a liquid trap filled with silica beads (Sigma Aldrich) and a mass flow meter (Bronkhorst), before it entered the GC. The GC was equipped with a pump at the outlet, which pumps in a constant amount of gas into the channels of the GC before a sample is taken, otherwise the gas is vented off. This allows the use of a larger gas flow without building up pressure in the lines.

The gas flow going into the GC is a mixture of the products, unreacted CO_2 and the argon carrier gas. The MFC used was calibrated for CO_2, hence the measured flow (Q_m) had to be corrected for the actual flow (Q_r) by using the gas conversion factors (GCF_i) for each separate component.

$$GCF_i = \frac{\rho_{N_2} \cdot Cp_{N_2}}{\rho_i \cdot Cp_i} \quad (1)$$

Then, the GCF for the mixture (GCF_{mix}) was calculated and the actual flow (Q_r) can be determined.

$$GCF_{mix} = \left(\frac{c_i}{GCF_i} + \frac{c_{i+1}}{GCF_{i+1}} + \cdots + \frac{c_n}{GCF_n} + \frac{10^6 - (c_i + c_{i+1} + \cdots + c_n)}{GCF_{Ar}}\right)^{-1} \cdot 10^6 \quad (2)$$

$$Q_r = Q_m \cdot \frac{GCF_{mix}}{GCF_{CO_2}} \quad (3)$$

The corrected gas flow was used to calculate the faradaic efficiency (FE)

$$FE = \frac{n \cdot c_i \cdot F \cdot Q_r \cdot p}{R \cdot T \cdot I} \cdot 100\% \quad (4)$$

Gas Chromatograph Calibration method

The gas chromatograph (GC) uses 3 channels and two different detectors. All channels use Helium as carrier gas. The first channel has on main Rtx-1 column (15 m length, 0.32 mm ID) and an flame ionization detector (FID). This channel is used for the detection of hydrocarbons, of which a calibration is available for methane and ethylene. The second channel has a Carboxen 1010 pre-column (3 m length, 0.32 mm ID), a Carboxen 1010 main column (7 m length, 0.32 mm ID), and a thermal conductivity detector (TCD). On this channel, only CO2 is calibrated. Theoretically, CO can also be detected on this channel. However, due to the use of Argon as flush gas in the electrochemical setup, these peaks are overlapping. The third channels uses a Rt-Qbond pre-column (3 m length, 0.32 mm ID), Molsieve 5A main column (7 m length, 0.32 mm ID), and a TCD as well. On this channel, hydrogen and CO are calibrated. The sensitivity for hydrogen is limited, therefore lower concentrations are less accurately detected. The amount of hydrogen that is produced in the electrochemical cell is in higher concentrations and therefore accurate for the measurements needed in this system.

The calibration is performed using 5 different gas bottles with fixed amounts of gases mixed in CO_2. The gas mixture is made up of hydrogen, carbon monoxide, methane, and ethylene. Each component is calibrated at a concentration of 50, 100, 1000, 3000, and 8000 ppm. CO_2 is calibrated separately, since this is the bulk gas in the calibration bottles. A back flush is used after the pre-column on the second and third channel to prevent the CO_2 from poisoning the main column. The CO2 calibration is performed by mixing N_2 and CO_2 in different concentrations using mass flow controllers (Bronkhorst). The chromatograms of for the different channels can be seen in Figure S3.

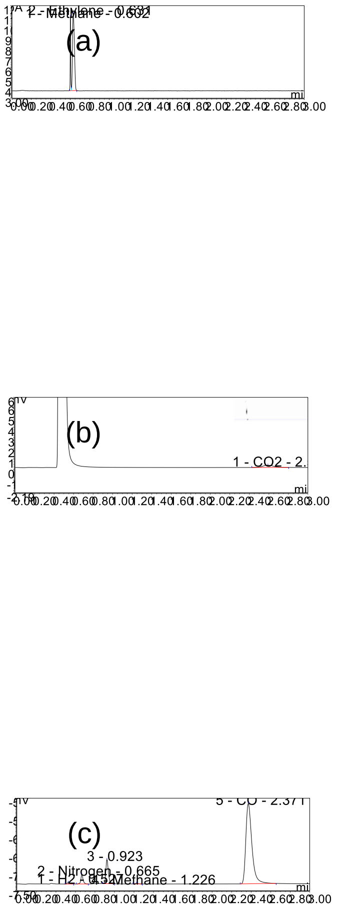

Figure S3. Chromatograms of (a) the first channel, measuring methane and ethylene, (b) the second channel, measuring CO2 and (c) the third channel, measuring hydrogen and CO.

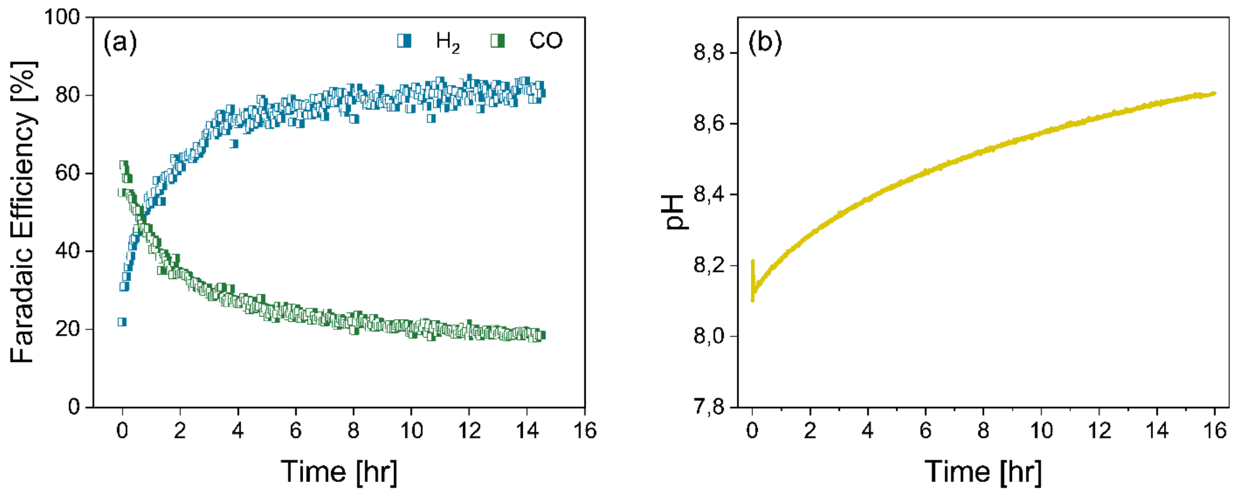

Figure S4. Longer term stability of zero-gap bicarbonate electrolyser at 100 mA/cm2 using a Ag spray-coated cathode and recirculating 3 M KHCO3 catholyte (1 L) and 1 M KOH anolyte (0.5 L). (a) Faradaic efficiencies over time towards H2 and CO for a zero-gap configuration. (b) The change in electrolyte bulk pH over time. All results are average values of duplicate measurements with an average error of ± 3% and total FE of >98%.

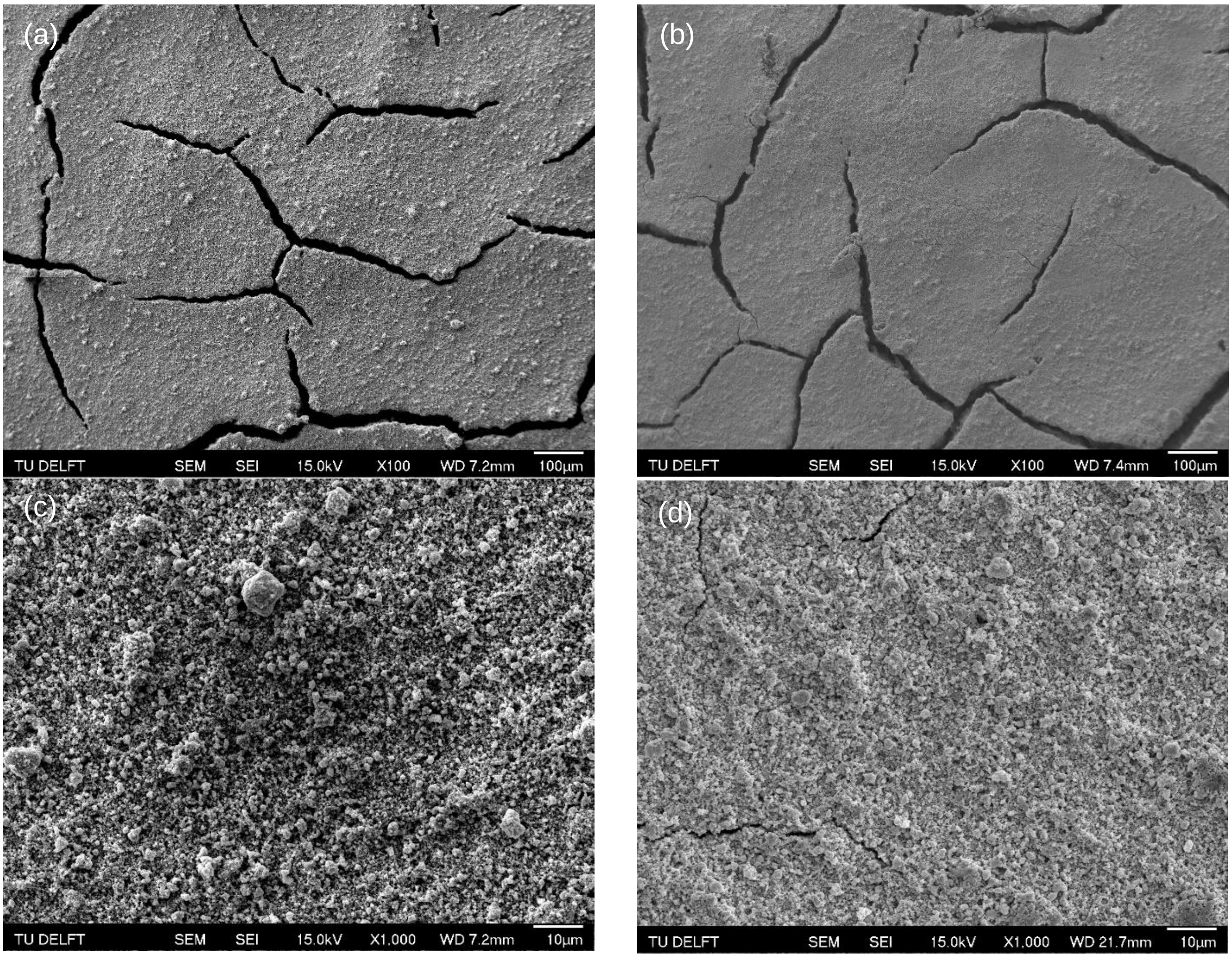

Figure S5. SEM images (a) and (c) fresh electrodes, (b) and (d) post-electrolysis. Except for small cracks due to the catholyte flow through the electrode, no visual changes of the surface are visible.

Table S1. ICP results for the electrolyte of the longer-term electrolysis runs (3 hours and 15 hours), presented in ppm.

**Sample**		**Ba**	**Ca**	**Fe**	**Na**	**Cl**
Blank		0.07	0.02	0.03	110.06	26.36
3hrs	Zero-gap	0.09	0.23	0.05	161.79	24.26
3hrs	135 μm	0.08	0.08	0.04	156.32	12.36
3hrs	270 μm	0.09	0.08	0.08	144.76	11.18
3hrs	405 μm	0.08	0.14	0.10	146.32	16.32
15hrs	Zero-gap	0.06	0.03	0.04	124.87	18.30
15 hrs	135 μm	0.07	0.03	0.05	108.70	4.95

Table S2. List of contaminants present in the $KHCO_3$ (batch BCCL2557) and K_2CO_3 (batch BCCL0256) used.[1, 2]

**Component**	**Concentration [mg/kg]**	
	$KHCO_3$ ≥ 99.7%	K_2CO_3 ≥ 99.0%
Sodium (Na)	< 300	< 200
Silicate (as SiO_2)	n.a.	< 50
Sulfur as SO_4	< 30	< 40
Calcium (Ca)	< 20	< 10
Magnesium (Mg)	< 10	< 5
Chloride (Cl)	< 10	< 10
Iron (Fe)	< 5	< 5
Phosphate (PO4)	< 5	< 10
Ammonia (NH_4)	< 5	n.a.
Heavy metals (as Pb)	< 5	< 5

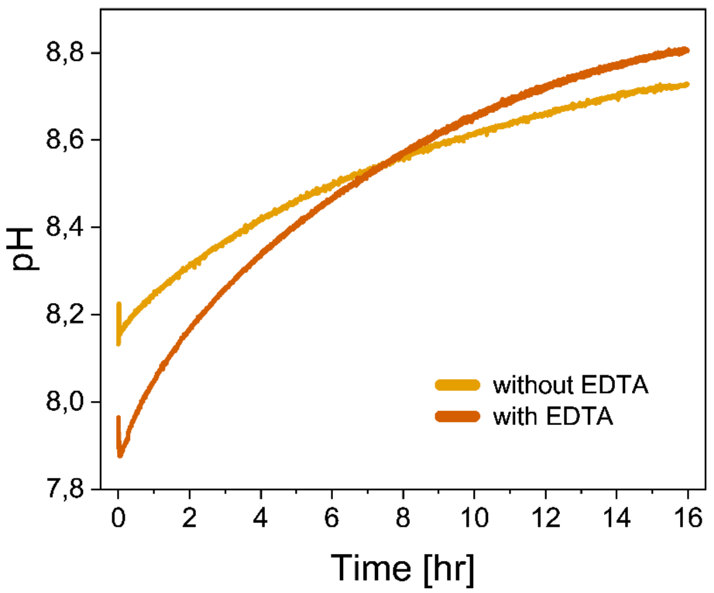

Figure S6. The change in electrolyte bulk pH over time for the longer term stability experiment of the bicarbonate electrolyser at 100 mA/cm2 using a Ag spray-coated cathode and recirculating 3 M KHCO3 catholyte (1 L) and 1 M KOH anolyte (0.5 L).

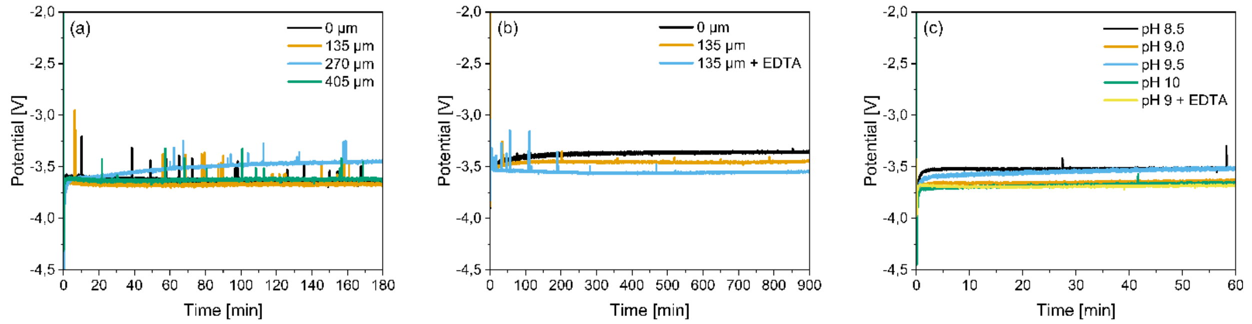

Figure S7. The measured potential over time for all (a) 3 hour experiments, (b) 15 hour experiments, and (c) single pass experiments.

References

[1] R. Schwenninger, Sigma-Aldrich, 2024.
[2] R. Schwenninger, Sigma-Aldrich, 2024.
